# Chinese medicine Gushukang capsule for treating primary osteoporosis: a systematic review and meta-analysis

**DOI:** 10.1186/s13018-023-04264-9

**Published:** 2023-11-08

**Authors:** Tianpeng Liu, Menglin Yao, Yifan Zhao, Shaochuan Zhao, Chen Rui, Feng Yang

**Affiliations:** 1 Shaanxi University of Chinese Medicine, Xianyang, 712046 China; 2 Southwest Medical University, Luzhou, 646000 China; 3 Affiliated Hospital of Shaanxi University of Chinese Medicine, Xianyang, 712000 China

**Keywords:** Primary osteoporosis, Gushukang capsules, Systematic review, Bone metabolism

## Abstract

**Objective:**

To systematically evaluate the efficacy and safety of Gushukang (GSK) capsules in the treatment of primary osteoporosis.

**Methods:**

Randomized controlled trials related to the treatment of primary osteoporosis were collected through online retrieval of the China National Knowledge Infrastructure (CNKI), Wanfang database, Chinese Biomedical Literature Database (Sino-Med), VIP, US National Library of Medicine (PubMed), Web of Science and Cochrane library. The literature was searched from January 1, 2000, to March 17, 2022. The risk bias and quality of the trials included in the meta-analysis were evaluated with the Cochrane Collaboration's risk assessment tool. The effect size was expressed as risk ratios (RRs) or mean differences (MDs) with 95% confidence intervals (CIs).

**Results:**

A total of 24 randomized controlled clinical trials (RCTs) were incorporated into this systematic review. The 2363 patients were all primary osteoporosis patients, of whom 1197 were in the observation group and 1166 were in the control group. GSK capsule group was superior to conventional medication group in improving beta type I collagen carboxy-terminal peptide (β-CTX) (MD − 0.28, 95% CI [− 0.31, − 0.25]), while in improving prepeptide of type I procollagen (PINP), conventional medications group was superior to GSK capsule group (MD − 1.37, 95% CI [− 1.92, − 0.82]), and there were no significant differences between the two groups in overall efficacy (OE) (OR 1.62, 95% CI [0.89, 2.98]), increase of bone mineral density (BMD) (lumbar spine: MD − 0.02, 95% CI [− 0.08, 0.04]; femoral neck: MD − 0.01, 95% CI [− 0.07, 0.05]; hip: MD 0.01, 95% CI [− 0.02, 0.02]), enhancement of alkaline phosphatase (ALP) (MD − 1.37, 95% CI [− 13.29, 10.55]), serum calcium (S-Ca) (MD 0.02, 95% CI [− 0.13, 0.17]), bone glutamyl protein (BGP) (MD 3.75, 95% CI [− 12.26, 19.76]), safety (OR 0.37, 95% CI [0.07, 2.02]) and pain relief (MD 0.32, 95% CI [− 0.59, 1.22]). GSK capsule combined with conventional medications group was superior to conventional medications group in improvement of OE (OR 3.19, 95% CI [2.20, 4.63]), BMD (lumbar spine (MD 0.06, 95% CI [0.02, 0.10]), femoral neck (MD 0.08, 95% CI [0.03, 0.13]), hip (MD 0.14, 95% CI [0.08, 0.21]) and other parts (MD 0.04, 95% CI [0.03, 0.05]), ALP (MD − 5.56, 95% CI [− 10.08, − 1.04]), β-CTX (MD − 0.15, 95% CI [− 0.18, − 0.12]) and pain relief (MD − 1.25, 95% CI [− 1.83, − 0.68]), but there was no difference in S-Ca (MD 0.02, 95% CI [− 0.13, 0.17]), BGP (MD 1.30, 95% CI [− 0.29, 2.89]), PINP (MD 1.30, 95% CI [− 0.29, 2.89]), serum phosphorus (S-P) (MD 0.01, 95% CI [− 0.09, 0.12]) and safety (OR 0.71, 95% CI [0.38, 1.35]).

**Conclusion:**

GSK capsules can effectively treat primary osteoporosis, and when combined with conventional medications, the drug significantly increased bone mineral density, relieved pain and improved bone metabolism-related indicators in primary osteoporosis patients with better efficacy. However, due to the inclusion of Chinese literature and possible publication bias, the reliability of conclusions still requires more high-quality RCTs to enhance.

**Supplementary Information:**

The online version contains supplementary material available at 10.1186/s13018-023-04264-9.

## Introduction

Primary osteoporosis (POP) is a bone metabolic disorder that is characterized by decreased bone mass and destruction of bone tissue microstructure, leading to increased bone fragility and fracture risk [1]. POP is generally divided into three categories: postmenopausal osteoporosis (type I), age-related osteoporosis (type II) and idiopathic osteoporosis. Type I and type II are the most common types of primary osteoporosis [2]. It was predicted that by the year 2050, 25% of China’s population will be over the age of 60 years old, and the number of POP patients will reach 212 million [3]. Furthermore, the number of POP-related fractures will also increase dramatically in the coming decades [4].

The main therapies for primary osteoporosis include physical exercise, nutritional supplements and anti-osteoporosis drugs, and medication is the most recommended treatment [5, 6]. It has been found that physical exercise in patients with POP could improve their BMD, strength, agility, and quality of life and reduce the risk of falling [7]. Moreover, traditional Chinese exercise, such as Ba Duan Jin, was helpful in improving BMD, improving balance and relieving pain in patients with POP [8]. A recent narrative review found that compared to taking vitamin D supplements alone, simultaneous supplementation with vitamin D and calcium was more effective in improving BMD [9]. Furthermore, calcium carbonate D_3_ combined with nutritional supplementation could improve POP patients’ OE, BMD and bone metabolism [10]. Current FDA-approved pharmacologic therapies and drugs for osteoporosis include bisphosphonates (e.g. alendronate), estrogen-related therapy (e.g., raloxifene conjugated estrogens), parathyroid hormone analogs (teriparatide), receptor activator of nuclear factor-κ B ligand (RANKL) inhibitor (e.g. denosumab), sclerostin inhibitor (e.g. romosozumab) and calcitonin salmon [11]. Specifically, for age-related osteoporosis, orthopedics-geriatrics co-management, appropriate weight training and timely surgery were suggested recently [12]. For postmenopausal osteoporosis, biomarkers of bone turnover, such as ALP, PINP and β-CTX, might play a role in predicting the prognosis of osteoporosis [13, 14]. Among denosumab, pamidronate and zoledronate, denosumab was found to obviously influence the BMD of the hip and femur and improve the BMD of the spine most obviously [15], and it was found that denosumab could significantly reduce nonvertebral fractures [16].

In recent years, *herbal medicine*, such as the traditional Chinese medicine GSK, has attracted the interest of medical researchers due to its low cost and few side effects. GSK consists of several traditional herbs, including *Longspur Epimedium* (Yinyanghuo), *Rhizoma Atractylodis* (Cangzhu), *Radix* *Astragali* (Huangqi) and *Rhizoma Drynariae* (Gusuibu) [17, 18]. Containing naringin and icariin, GSK could effectively stimulate the production of vitamin D [19]. Another study found that a bioactive compound, icariin, which could be isolated from *Epimedium koreanum* (Chaoxianyin Yang Huo), ameliorated estrogen deficiency-induced osteoporosis by promoting insulin-like growth factor 1 (IGF-I) signaling in bone [20]. Moreover, one study recognized the systematic bone protection of GSK by inhibiting osteoclast formation and stimulating osteoblast formation, laying the foundation for developing new drugs to treat POP [21]. According to traditional Chinese medicine, POP is caused by deficiency of the liver, spleen and kidney and stagnation of Qi and blood, so the treatment is based on warming the kidney and liver, strengthening the spleen and resolving blood stasis [22]. On the basis of this theory, discriminatory treatment often achieves good results with a high safety level [23]. GSK is a pure traditional Chinese medicine with the principle of tonifying the kidney and benefiting Qi, invigorating the blood and strengthening the bones [24].

Currently, most findings about GSK are positive, but the quality of some trials is not reliable enough, and there is not a systematic analysis for the drug thus far. As a result, we sought to systematically evaluate the efficacy and safety of GSK in treating POP with the aim of providing an evidence-based basis for the rational clinical use of the drug in the prevention and treatment of POP.

## Methods and materials

The meta-analysis was conducted on the basis of the PRISMA 2020 guidelines [25]. The protocol of the meta-analysis has been registered at the International Platform of Registered Systematic Review and Meta-analysis Protocols (INPLASY) (Registration number: INPLASY202370023) and is available in full on inplasy.com (https://inplasy.com/inplasy-2023-7-0023/) (Additional file 1).

### Search strategy

We searched CNKI, VIP, Sino-Med, Wanfang database, PubMed, Cochrane library and Web of Science from their foundation to March 20th, 2023. The search terms were used individually or combined as follows: “osteoporosis”, “bone loss”, “bone disease”, “post-traumatic osteoporosis”, “senile osteoporosis”, “age-related osteoporosis”, “postmenopausal osteoporosis”, “Gushukang” and “randomized controlled trial”. Chinese search terms included “guzhishusong”, “Gushukang”, and “suijiduizhaoshiyan”.

To improve the completeness of the literature search, we adapted the search strategy to the different characteristics of the databases and thus performed a comprehensive search. The search strategy for PubMed is shown in the “Appendix” at the end of the paper.

### Inclusion and exclusion criteria

#### Inclusion criteria

(1) Study design: randomized controlled trials in all languages, and blinding was needed; (2) study population: patients diagnosed with primary osteoporosis [26]; (3) intervention: the observation groups were treated with GSK or GSK combined with conventional medications, while the control groups were treated with conventional medications.

#### Exclusion criteria

(1) Original paper: duplicate studies or types of literature such as reviews, editorials, letters, notes and statements. (2) Trial subjects: laboratory studies or animal experiments. (3) Trial type: nonrandomized controlled trials. (4) Intervention: GSK capsules were used in the control group. (5) Trial outcome: data missing or obviously incorrect.

### Outcome measures

#### Primary outcomes

(1) OE, the calculation formula was (Effective patients’ number/Total patients’ number) * 100%; (2) BMD, measured by dual energy X-ray bone densitometry, but the brand of the testing instrument may vary from different hospitals; (3) Visual analog score (VAS).

#### Secondary outcomes

(1) PINP; (2) β-CTX; (3) BGP; (4) S-P; (5) S-Ca; (6) ALP; (7) Adverse reactions (AE).

### Study selection

Two authors independently screened the titles and abstracts of all literature collected and reviewed the studies for eligibility according to the inclusion and exclusion criteria, with another researcher being consulted in the event of disagreement over the ranking of a particular piece of literature.

### Data extraction

Two researchers (TP Liu and YF Zhao) extracted data independently (including OE, BMD, ALP, VAS score, S-Ca, S-P, BGP, β-CTX, PINP and AE) with a data form made by Microsoft Excel 2021. The extracted data were checked, and any disagreements were discussed and resolved with F Yang.

### Risk of bias assessment

Two researchers (T-PL and M-LY) assessed the risk of the trials included with the Cochrane Collaboration's risk of bias assessment tool [27], which was assessed on six main items: (1) random allocation method; (2) allocation concealment scheme; (3) blinded implementation; (4) completeness of outcome data; (5) selective reporting of study results; and (6) other sources of bias issues to determine the level of risk of bias in the studies. If there were disagreements, a third author (FY) was invited into the discussion to determine the risk.

### Data analysis and synthesis

Review Manager (version: 5.4.1) was chosen to analyze the data. Heterogeneity was tested by the I^2^ value of those trials. A fixed-effects model was applied to statistical analysis if there was no statistical heterogeneity among the trials (I^2^ ≤ 50%), while a random-effects model was used when high heterogeneity was proven (I^2^ > 50%). Inverted funnel plot analysis was conducted for publication bias. The two dichotomous variables, OE and adverse effects, were analyzed with the odds ratio (OR) with 95% confidence intervals, while the remaining continuous variable outcome indicators (BMD, VAS score, PINP, β-CTX, ALP, S-Ca, S-P and BGP) were analyzed with the mean difference (MD) and 95% confidence intervals (CI). Specifically, considering that BMD may vary in different parts of the skeletal system, subgroup analysis was performed by area, including the lumbar spine, femoral neck, hip and other parts (greater trochanter of femur, trochanter of femur and Ward’s triangle).

## Results

### Study selection

**Fig. 1:**
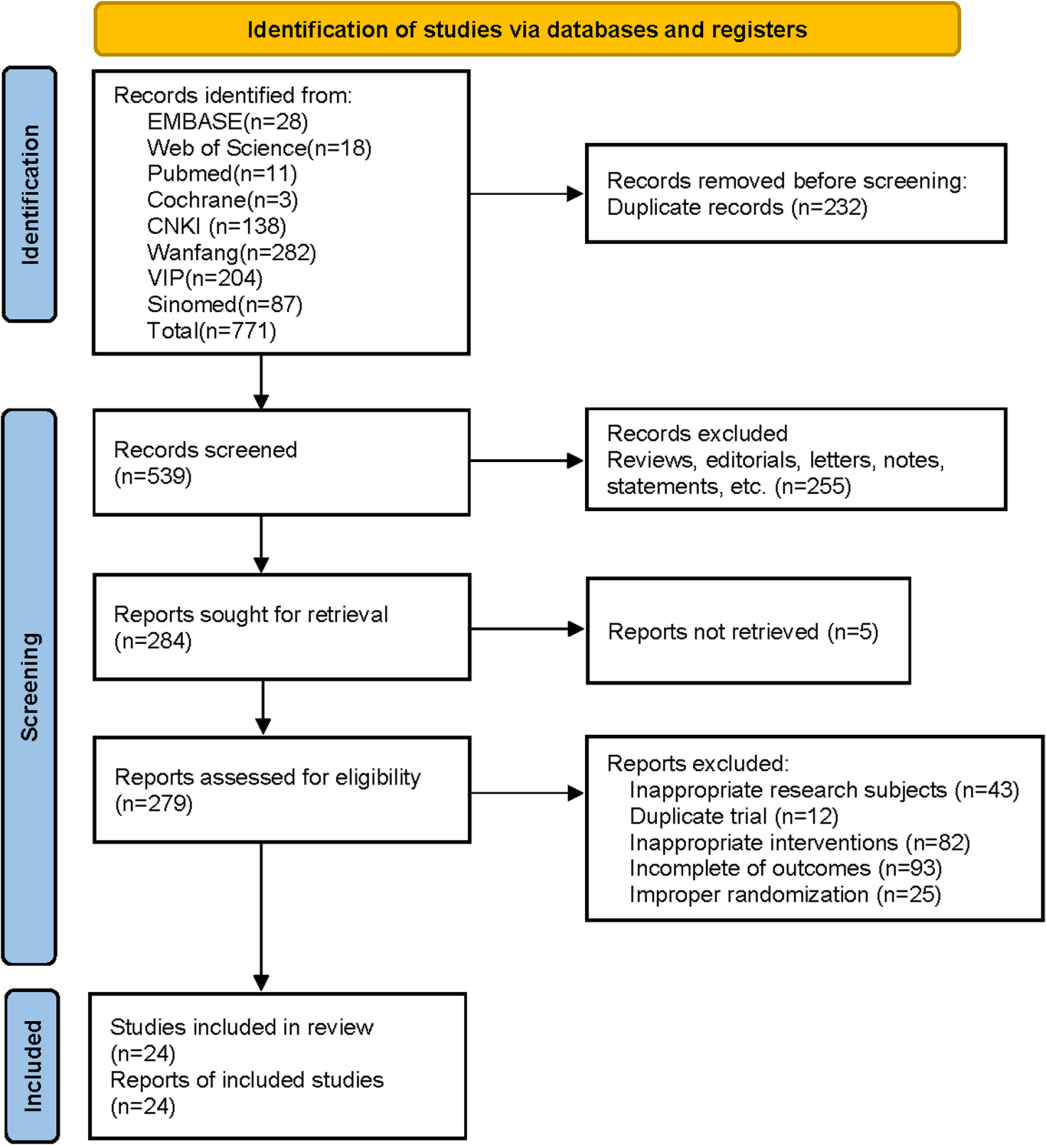
Flowchart of the study selection process

A total of 771 papers were retrieved according to the established search strategy, including 711 articles in Chinese and 60 articles in English. By scanning the titles and abstracts, 232 duplicates were excluded, and through further checking of the full text, 515 of them did not meet the inclusion criteria and were excluded. The flowchart (Fig. 1) with the number of included studies at each step was established, including reasons for excluding studies. Twenty-four trials were finally included. The flow chart (Fig. 1) was developed below, listing the number of studies included at each step, including reasons for excluding studies. Twenty-four trials were ultimately included.

### Study characteristics

A total of 24 RCTs were included, and all 2363 cases included were patients with primary osteoporosis, of which 1197 were observations and 1166 were controls. The maximum sample size of individual studies was 98, and the minimum sample size was 19. Nine [28–36] studies found adverse reactions, and one [29] specified no adverse reactions. The conventional treatments in the control groups were conventional medications, including calcium D, alfacalcidol, nylstilbestrol, alendronate sodium, vitamin D and salmon calcitonin, as well as the combination of some of them. Specific information on the trials included in this study is shown in Table 1.

**Table 1:** Characteristics of randomized controlled trials on GSK capsule for osteoporosis

Study	Sample size (*T*/*C*)	Age (years old)		Medical condition	Intervention		Duration (months)	Outcomes	Adverse events
		*T*	*C*		*T*	*C*			
Bai et al. [37]	58/57	65.5 ± 10.5	65.9 ± 11.2	Postmenopausal osteoporosis	GSK 10 g Bid + alfacalcidol 1 μg Qd	Alfacalcidol 1 μg Qd	9	④	Not mentioned
Chen 1 [38]	23/19	62.53 ± 9.24	63.21 ± 10.1	Postmenopausal osteoporosis	GSK 10 g Bid + calcium carbonate 750 mg and vitamin D_3_ 100 IU Bid	Calcium carbonate 750 mg and vitamin D_3_ 100 IU Bid	6	⑥⑧④⑨	Not mentioned
Chen [28]	45/45	53.4 ± 6.6	46.5 ± 8.5	Senile osteoporosis	GSK 10 g Bid + rocaltrol 0.25 μg Qd + alendronate sodium 70 mg Qw	Rocaltrol 0.25 μg Qd + alendronate sodium 70 mg Qw	6	⑦⑤⑧④	Mentioned
Cheng et al. [39]	80/80	67.07 ± 5.59	67.54 ± 5.81	Senile osteoporosis	GSK 10 g Bid + calcium carbonate 750 mg and vitamin D_3_ 100 IU Bid	Calcium carbonate 750 mg and vitamin D_3_ 100 IU Bid	6	⑥⑨⑧④	Not mentioned
Cong et al. [29]	53/53	62.24 ± 10.36	62.58 ± 10.66	Senile osteoporosis	GSK 1.28 g Bid + rocaltrol 0.25 μg Qd + alendronate sodium 70 mg Qw	Rocaltrol 0.25 μg Qd + alendronate sodium 70 mg Qw	6	⑦⑥⑧⑤④	Not found
Feng et al. [40]	35/31	72.6 ± 16.4	72.3 ± 14.7	Senile osteoporosis	GSK 10 g Bid + salcatonin 100 IU Qod + calcium carbonate 750 mg and vitamin D_3_ 100 IU Tid	Salcatonin 100 IU Qod + calcium carbonate 750 mg and vitamin D_3_ 100 IU Tid	6	⑦	Not mentioned
Guo et al. [41]	39/39	62.3 ± 3.7	62.7 ± 3.2	Postmenopausal osteoporosis	GSK 10 g Bid + alfacalcidol 0.5 μg Qd	Alfacalcidol 0.5 μg Qd	6	⑥④	Not mentioned
Li [42]	55/40	61.5 ± 12.5	61.7 ± 13.7	Senile osteoporosis	GSK 10 g Tid + calcium carbonate 600 mg and vitamin D_3_ 125 IU Tid + salcatonin 20 μg Qod	Calcium carbonate 600 mg and vitamin D_3_ 125 IU Tid + salcatonin 20 μg Qod	6	⑦	Not mentioned
Li [30]	60/60	56.3 ± 2.5	56.1 ± 2.4	Postmenopausal osteoporosis	GSK 10 g Bid + vitamin D_3_ 100 IU and salcatonin 100 IU Qd	Vitamin D_3_ 100 IU and salcatonin 100 IU Qd	6	⑦	Mentioned
Li [43]	44/41	70.21 ± 10.21	71.27 ± 10.35	Senile osteoporosis	GSK 10 g Tid + calcium carbonate 1.5 g and vitamin D_3_ 125 IU Bid	Calcium carbonate 1.5 g and vitamin D_3_ 125 IU Bid	3	⑦	Not mentioned
Li [44]	40/40	68.3 ± 5.7	68.5 ± 5.4	Senile osteoporosis	GSK GSK 1.28 g Bid + vitamin D_3_ 100 IU Bid	Vitamin D_3_ 100 IU Bid	3	⑦⑥①	Not mentioned
Lin et al. [31]	48/48	71.42 ± 3.85	71.25 ± 3.96	Senile osteoporosis	GSK 1.28 g Bid + alfacalcidol 0.5 μg Qd	Alfacalcidol 0.5 μg Qd	6	⑦⑥	Mentioned
Lu et al. [45]	40/40	68.3 ± 12.3	68.1 ± 11.2	Postmenopausal osteoporosis	GSK 12 g Tid	Nilestriol 2 mg Biw	6	⑦④⑥	Not mentioned
Ren [46]	35/30	60.21 ± 2.35	61.37 ± 1.26	Senile osteoporosis	GSK 10 g Bid + alendronate sodium 70 mg Qw	Alendronate sodium 70 mg Qw	3	⑥①	Not mentioned
Shen [32]	40/40	61.28 ± 11.7	62.12 ± 11.91	Senile osteoporosis	GSK 1.28 g Bid + calcium carbonate 750 mg and vitamin D_3_ 100 IU Qd	Calcium carbonate 750 mg and vitamin D_3_ 100 IU Qd	6	⑧⑥⑤	Mentioned
Shi [33]	70/70	54.3 ± 6.1	55.5 ± 6.9	Postmenopausal osteoporosis	GSK 1.28 g Bid + salcatonin 20 μg and estradiol valerate 1 mg Qd	Salcatonin 20 μg and estradiol valerate 1 mg Qd	6	⑦①⑥	Mentioned
Shi [47]	45/48	62.91 ± 2.89	62.84 ± 2.33	Senile osteoporosis	GSK 10 g Bid	Alendronate sodium 70 mg Qw	6	⑧④	Not mentioned
Wang [48]	35/35	69.04 ± 3.04	68.26 ± 2.74	Senile osteoporosis	GSK 10 g Qd	Alfacalcidol 0.5 μg Qd	6	⑧⑥⑤	Not mentioned
Wu [49]	70/70	58.2 ± 4.5	58.6 ± 4.7	Postmenopausal osteoporosis	GSK 10 g Qd + estrogen 0.625 mg Qd + vitamin D_3_ 100 IU Qd	Estrogen 0.625 mg Qd + vitamin D_3_ 100 IU Qd	12	⑥④	Not mentioned
Yuan et al. [50]	42/42	67.46 ± 7.89	67.38 ± 7.07	Postmenopausal osteoporosis	GSK 1.28 g Bid + rocaltrol 0.25 µg and calcium carbonate D_3_ 500 mg Qd	Rocaltrol 0.25 µg Qd and calcium carbonate D_3_ 500 mg Qd	6	⑥④②③	Not Mentioned
Zhang [34]	40/40	62.9 ± 3.5	60.2 ± 4.8	Senile osteoporosis	GSK 10 g Tid	Calcium carbonate 750 mg and vitamin D_3_ 100 IU Tid + salcatonin 100 IU Qod	6	⑦	Mentioned
Zhang [35]	56/56	84.3 ± 6.3	85.2 ± 6.5	Senile osteoporosis	GSK 10 g Bid + alendronate sodium 70 mg Qw + rocaltrol 0.25 μg Qd + calcium carbonate 750 mg and vitamin D_3_ 100 IU Bid	Alendronate sodium 70 mg Qw + rocaltrol 0.25 μg Qd + calcium carbonate 750 mg and vitamin D_3_ 100 IU Bid	6	⑥①②③	Mentioned
Zhong [51]	46/46	54.2 ± 3.2	52.4 ± 2.1	Senile osteoporosis	GSK 12 g Tid + calcium carbonate 750 mg and vitamin D_3_ 100 IU Bid + salcatonin 20 µg Qod	Calcium carbonate 750 mg and vitamin D_3_ 100 IU Bid + salcatonin 20 µg Qod	6	⑦	Not mentioned
Zhou [36]	98/96	85.3 ± 1.6	85.7 ± 1.8	Senile osteoporosis	GSK 10 g Bid + C alendronate sodium 70 mg Qw + calcium carbonate 600 mg and vitamin D_3_ 125 IU Qd	Alendronate sodium 70 mg Qw + calcium carbonate 600 mg and vitamin D_3_ 125 IU Qd	12	⑦①⑥⑧④⑤	Mentioned

T, treatment group; C, control group; GSK, Gushukang capsule; Qd, once daily; Qod, once for every other day; Bid, twice daily; Tid, three times daily; g, gram; mg, milligram; μg, microgram; Qw, once per week; Biw, twice per week; ① VAS, visual analog scale; ② PINP, prepeptide of type I procollagen; ③ β-CTX, beta type I collagen carboxy-terminal peptide; ④ ALP, alkaline phosphatase; ⑤ BGP, bone glutamyl protein; ⑥ BMD, bone mineral density; ⑦ OE, overall efficiency; ⑧ S-Ca, serum calcium; ⑨ S-P, serum phosphorus

### Risk of bias of individual studies

**Fig. 2:**
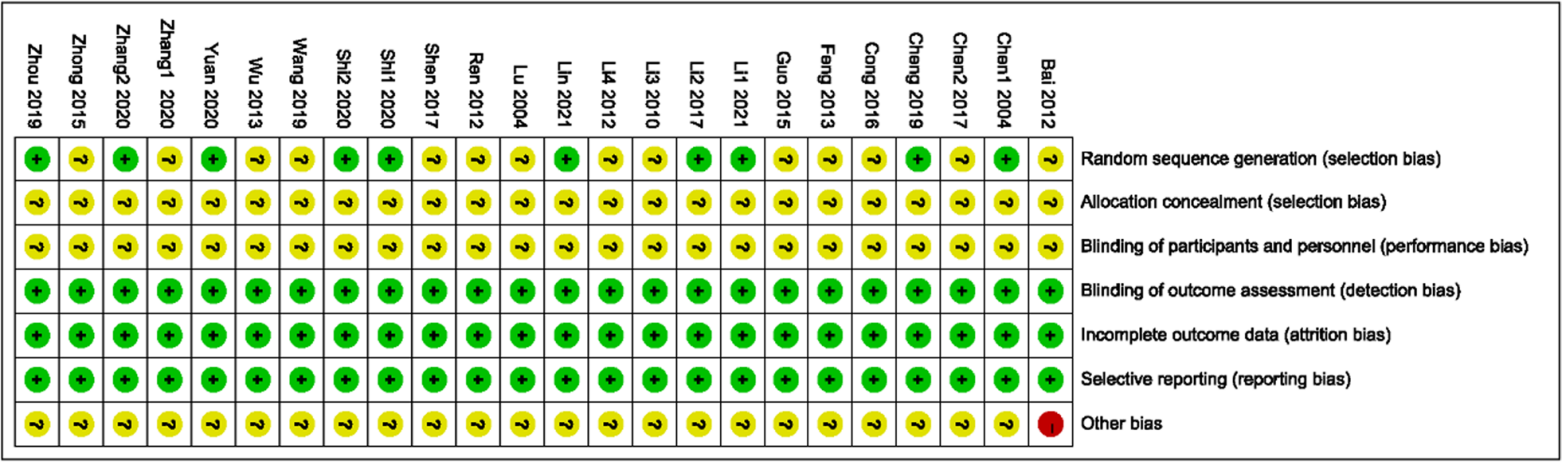
Risk of bias summary

**Fig. 3:**
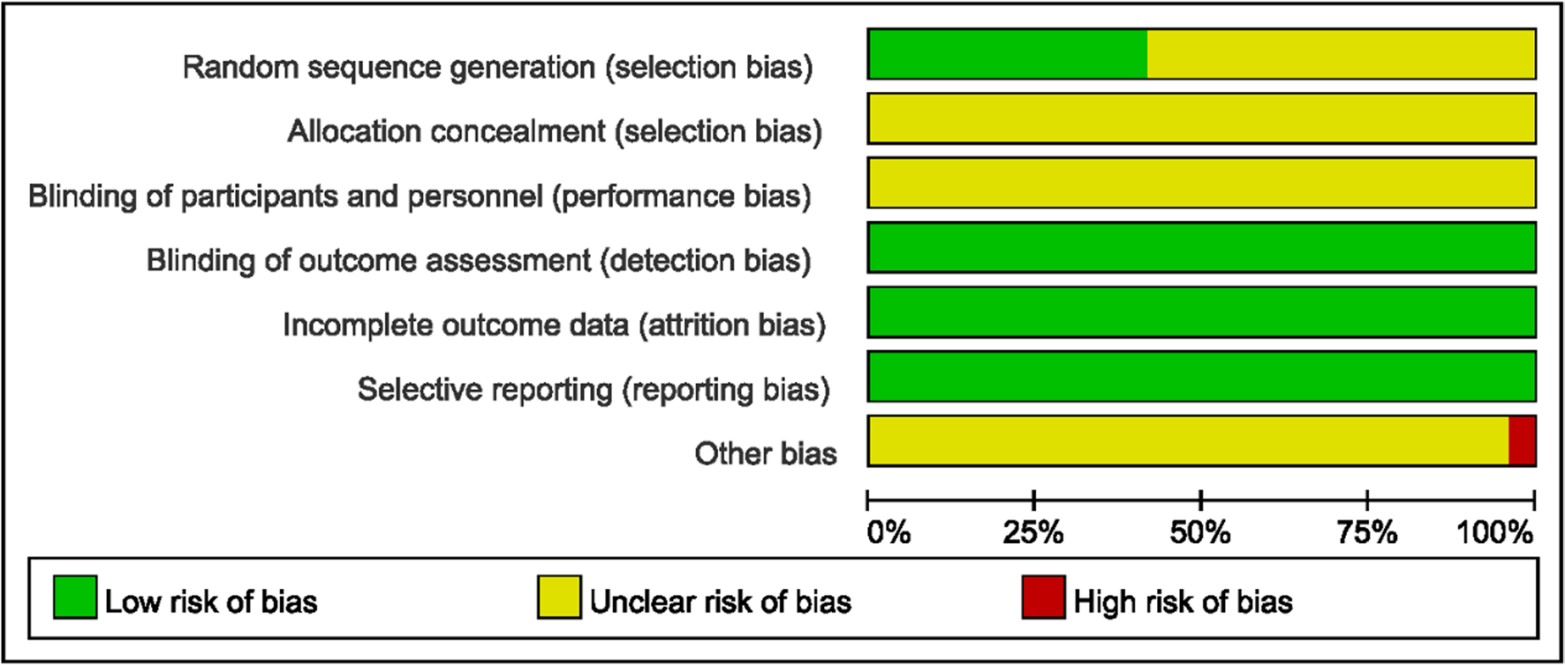
Risk of bias graph

Figures 2 and 3 were drawn to show each included study’s risk of bias. All twenty-four trials were grouped with a randomized method, of which 10 used a random number table method [30, 31, 33, 35, 36, 38, 39, 44, 47, 50] and the remaining fourteen did not describe a specific randomization method [28, 29, 32, 34, 37, 40–43, 45–47, 49, 51]. None of the allocation concealment schemes were described; none accounted for whether patients and investigators were blinded; and none accounted for whether outcomes were assessed. None described data completeness. All reported on prespecified indicators. None of the trials described sources of bias.

### Primary outcomes

#### Overall efficiency (OE)

**Fig. 4:**
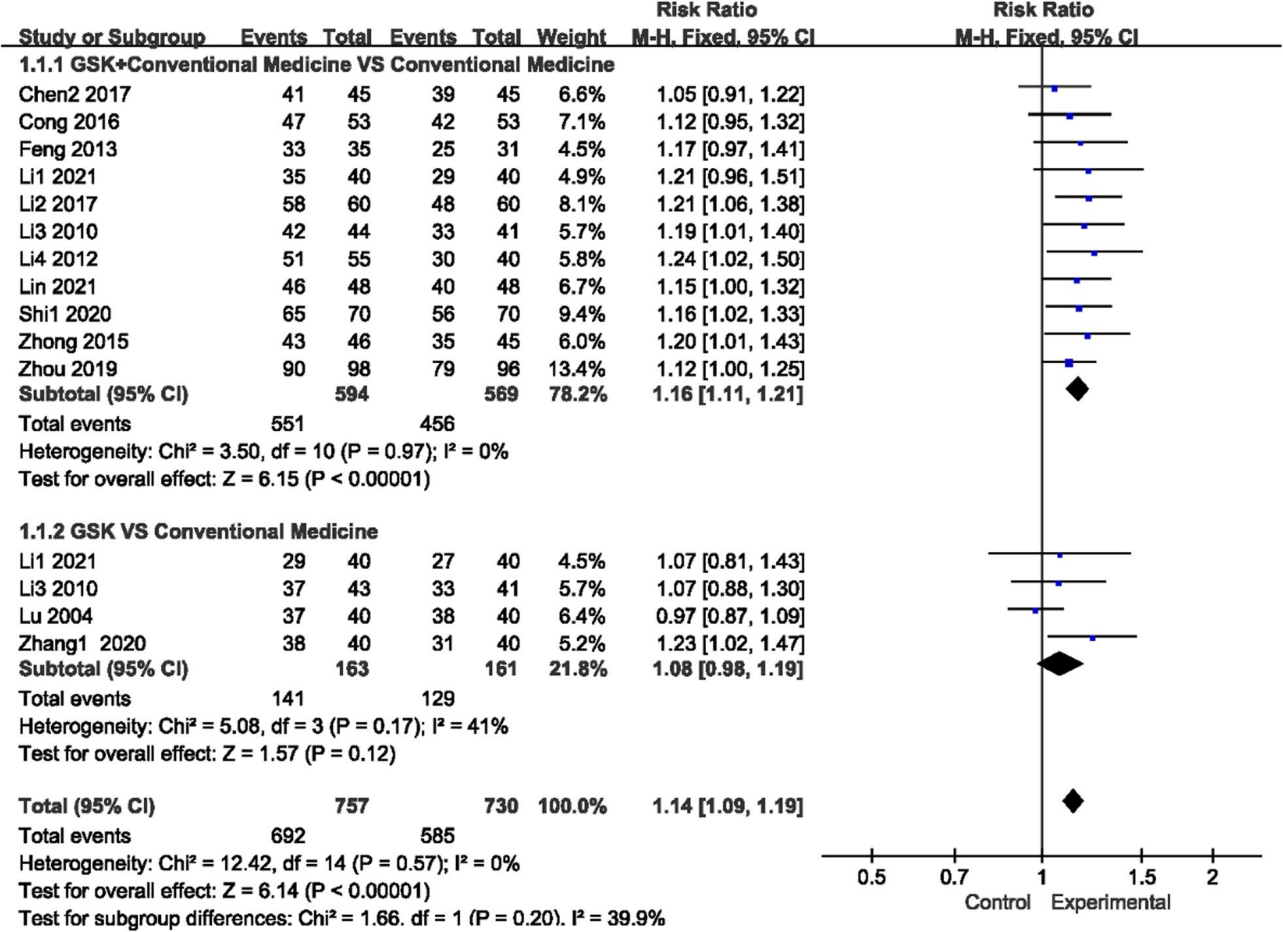
Forest plots of OE

Fifteen trials reported OE (Fig. 4), and there was no heterogeneity for those two comparisons (*P* = 0.97, I^2^ = 0%)/(*P* = 0.17, I^2^ = 41%), so a fixed-effects model was used to analyze the trials. GSK plus conventional medications (alendronate sodium, Caltrate D (containing calcium carbonate and vitamin D_3_), salcatonin, estradiol valerate, vitamin D, alfacalcidol and rocaltrol, used alone or in combination) were more effective than conventional medications (551/594 vs 456/569; RR 1.16, 95% CI [1.11, 1.21]). However, there was no obvious difference between GSK and conventional medications (141/163 vs 129/161; RR 1.08, 95% CI [0.98, 1.19]).

### Secondary outcomes

#### Bone mineral density

**Fig. 5:**
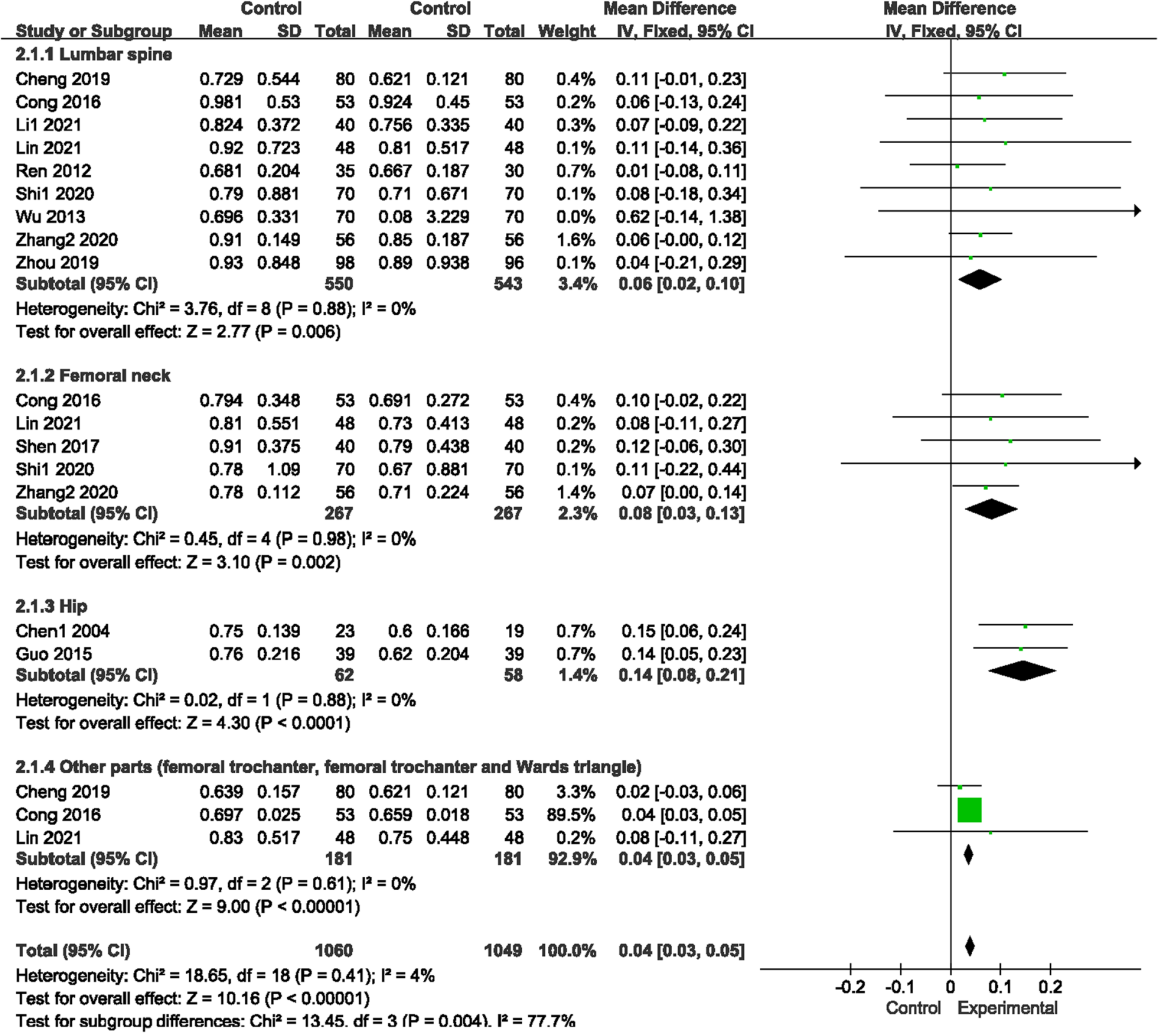
Forest plots of BMD (GSK + Conventional Medicine vs Conventional Medicine)

**Fig. 6:**
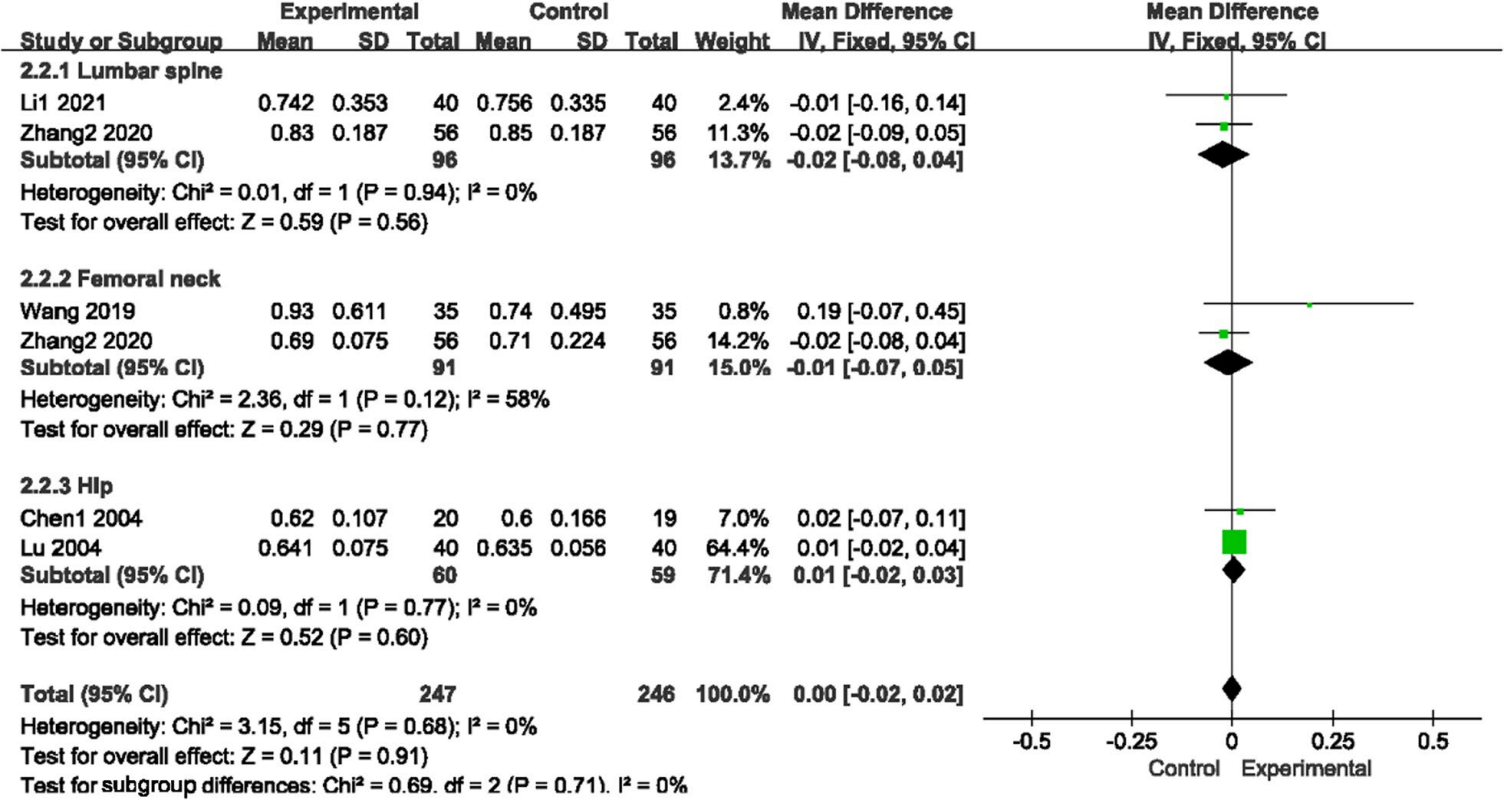
Forest plots of BMD (GSK vs conventional medicine)

Seventeen trials reported BMD (Figs. 5, 6), sorted by different areas, such as the lumbar spine, femoral neck, hip and other parts (femoral trochanter, femoral trochanter and Ward’s triangle). There was no heterogeneity for those two comparisons (*P* = 0.41, I^2^ = 4%)/(*P* = 0.94, I^2^ = 0%); thus, a fixed-effects model was used to analyze the trials. Compared with conventional medications, GSK plus conventional medications increased BMD levels in the lumbar spine (conventional medications include rocaltrol + alendronate sodium, alendronate sodium, salcatonin + estradiol valerate, alendronate sodium + Caltrate D, sodium + rocaltrol + Caltrate D, Caltrate D, vitamin D, alfacalcidol and estrogen + vitamin D) (MD 0.06, 95% CI [0.02, 0.10]), femoral neck (conventional medications include rocaltrol + alendronate sodium, salcatonin + estradiol valerate, alendronate sodium + rocaltrol + Caltrate D, alfacalcidol and Caltrate D) (MD 0.08, 95% CI [0.03, 0.13]), hip (conventional medications include alfacalcidol and Caltrate D) (MD 0.14, 95% CI [0.08, 0.21]) and other parts (conventional medications include alfacalcidol and Caltrate D) (MD 0.04, 95% CI [0.03, 0.05]). However, no variations were found in the contrast of GSK and conventional medications in those three areas (lumbar spine: MD [− 0.02, 95% CI [− 0.08, 0.04]; femoral neck: MD − 0.01, 95% CI [− 0.07, 0.05]; hip: MD 0.01, 95% CI [− 0.02, 0.02]).

#### Visual analog score (VAS)

**Fig. 7:**
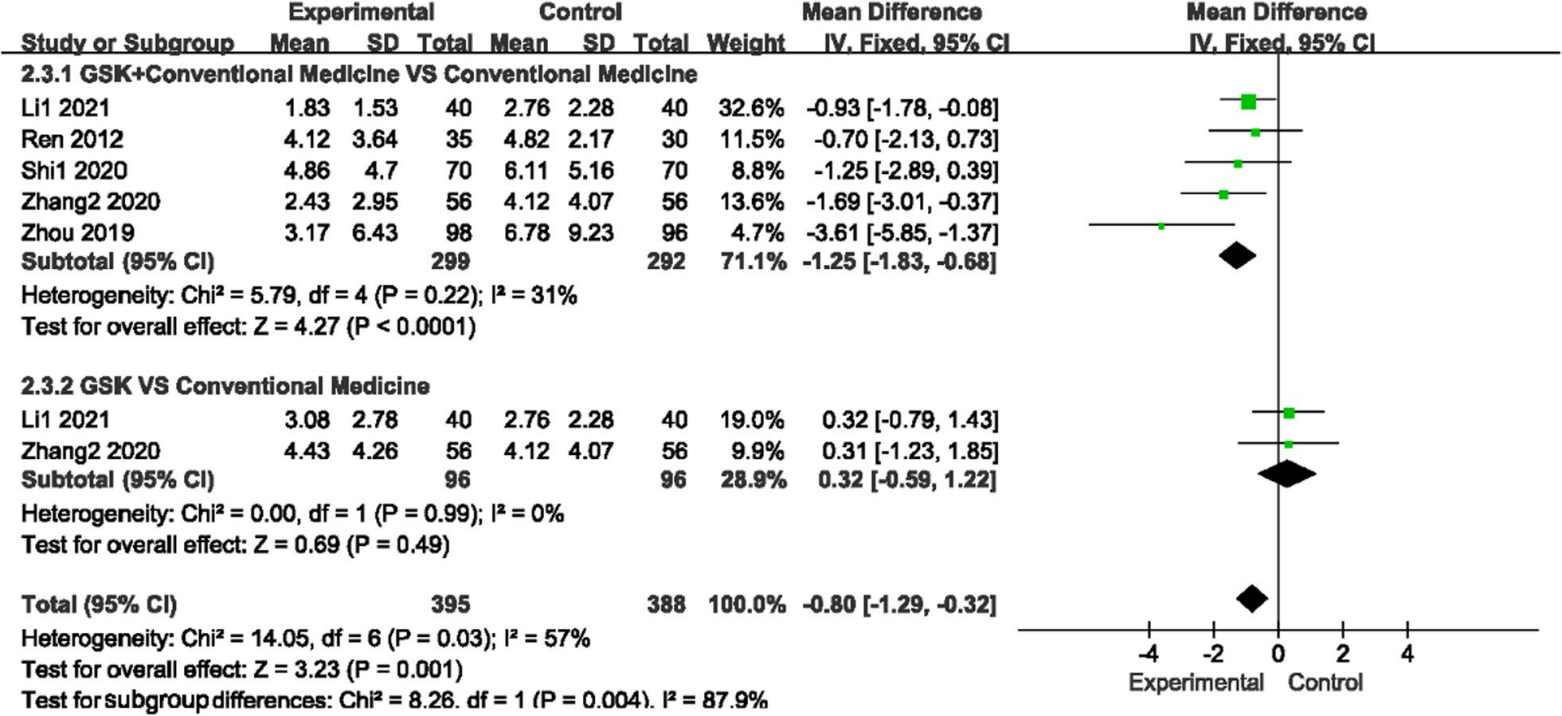
Forest plots of VAS scores

The VAS score (0 ~ 10 score) was used by seven studies to measure pain, as there was low heterogeneity for those two comparisons (*P* = 0.22, I^2^ = 31%)/(*P* = 0.32, I^2^ = 0%); a fixed-effects model was used to analyze the trials. Figure 7 shows that compared with conventional medications, GSK plus conventional medications (nilestriol, Caltrate D + salcatonin, vitamin D and Caltrate D) significantly relieved pain (MD − 1.25, 95% CI [− 1.83, − 0.68]). Compared to vitamin D or alendronate sodium + rocaltrol + Caltrate D, GSK alone did not show any advantage (MD 0.32, 95% CI [− 0.59, 1.22]).

#### Biochemical indicators

##### Serum calcium (S-Ca) and phosphorus (S-P) levels

**Fig. 8:**
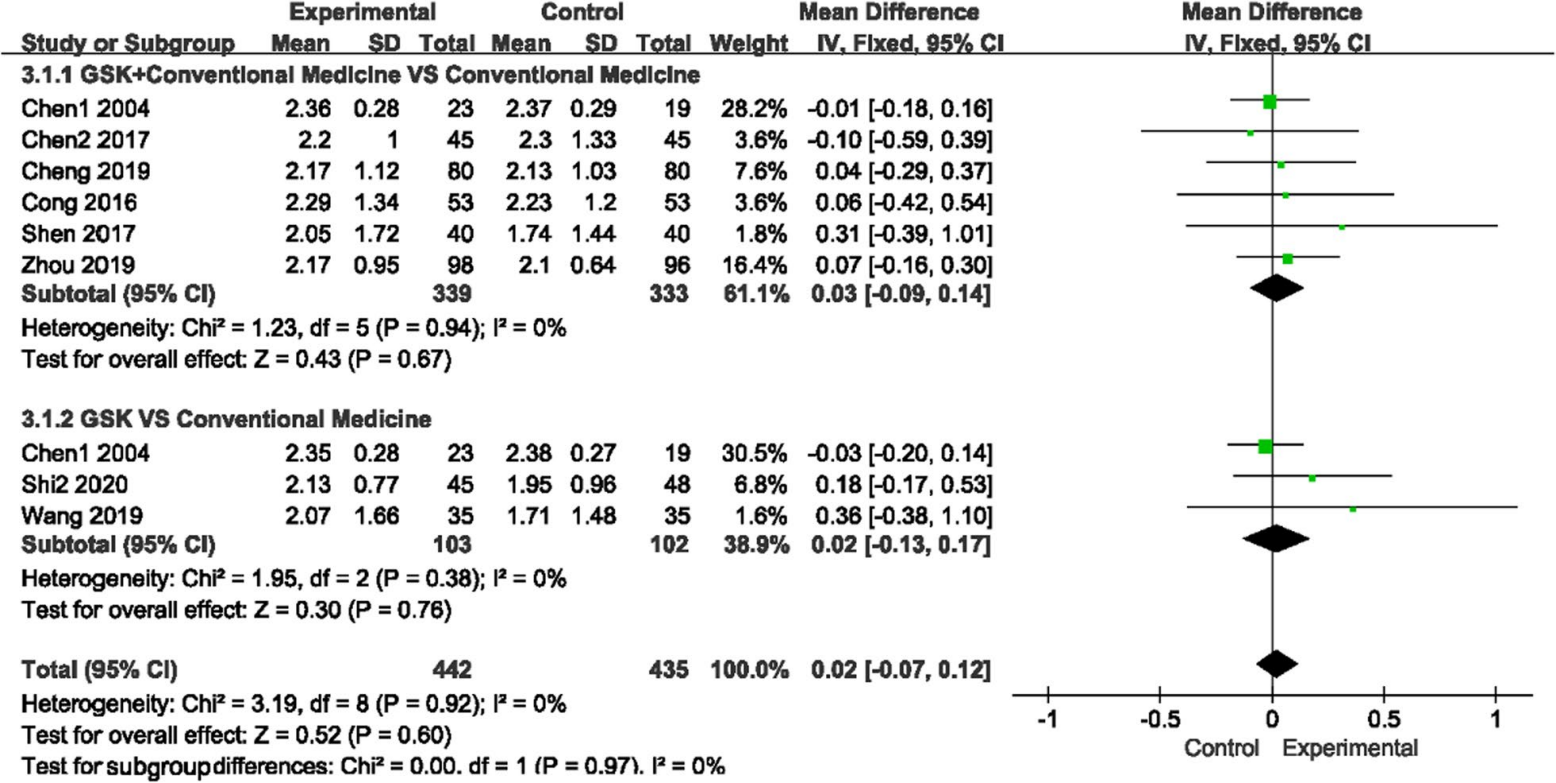
Forest plots of S-Ca level

**Fig. 9:**
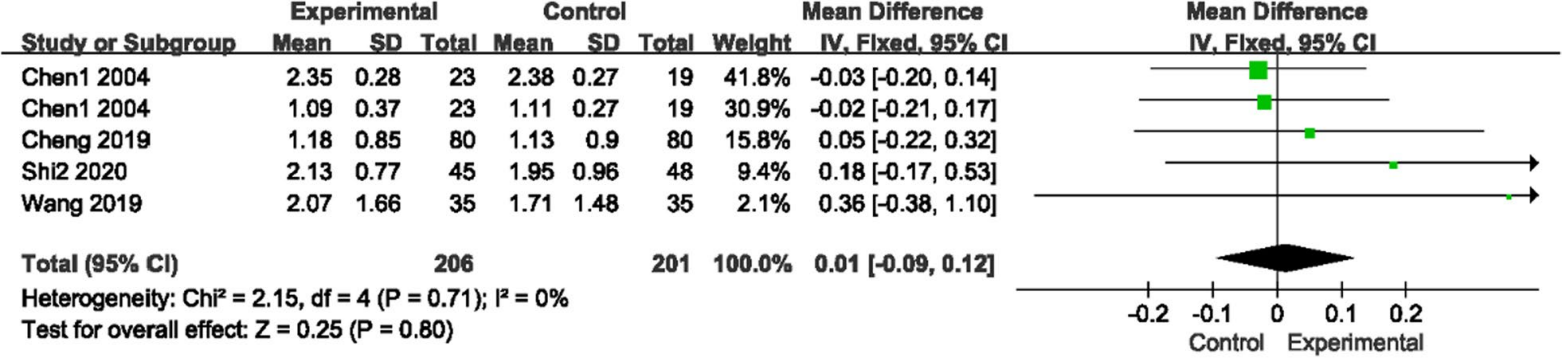
Forest plots of S-P level

For the two indicators, there was no heterogeneity in those trials, so a fixed-effects model was used to analyze the data (Figs. 8, 9). No comparison indicated a difference between GSK plus conventional medications and conventional medications in S-Ca (MD 0.03, 95% CI [− 0.09, 0.14]) and S-P (MD 0.01, 95% CI [− 0.09, 0.12]), yet in three trials, S-Ca levels in the GSK plus conventional medications (Caltrate D, alendronate sodium and rocaltrol) group were obviously higher than those in the conventional medications alone group.

##### Alkaline phosphatase (ALP) and beta type I collagen carboxy-terminal peptide (β-CTX) levels

**Fig. 10:**
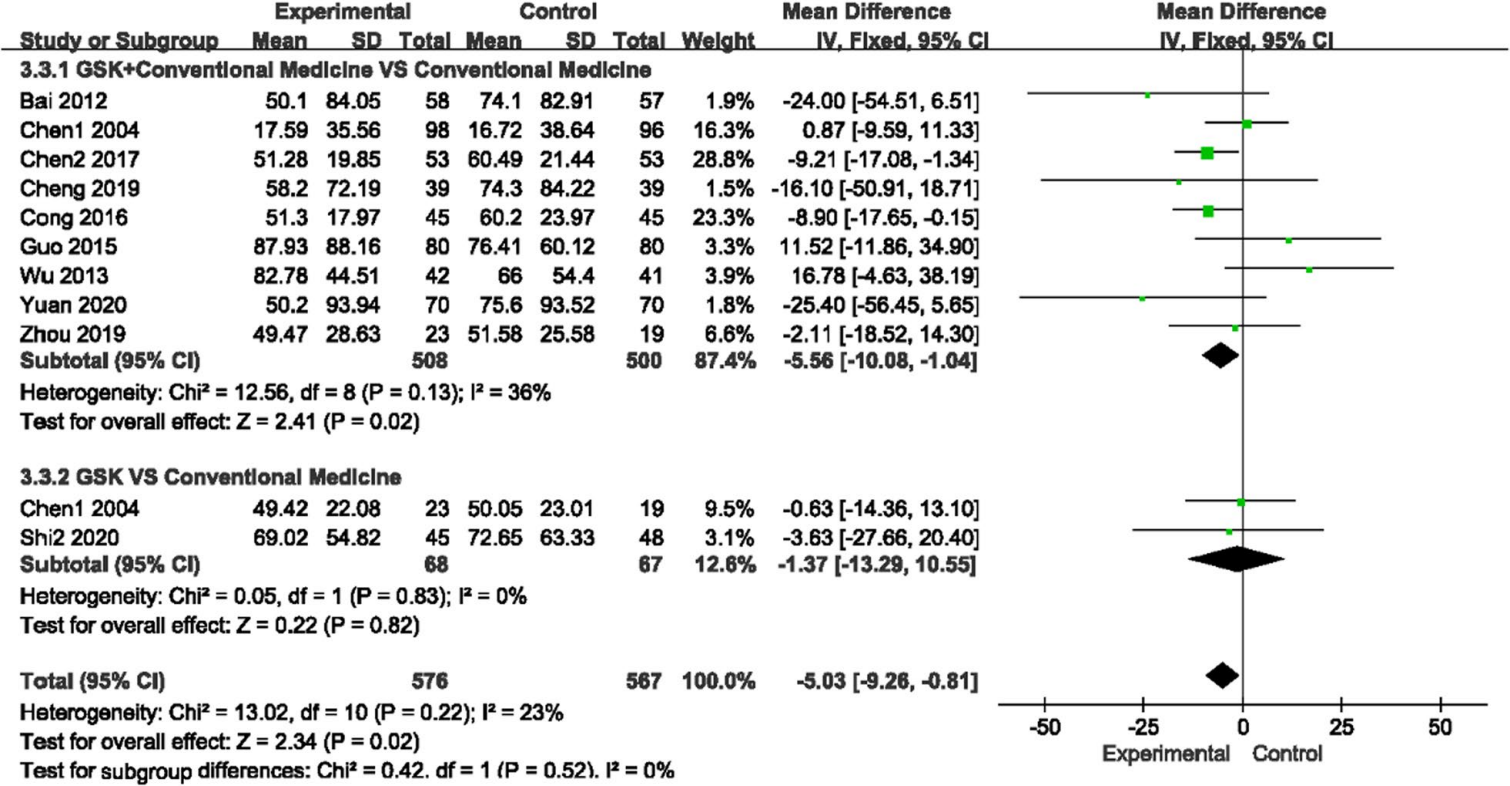
Forest plots of ALP levels

**Fig. 11:**
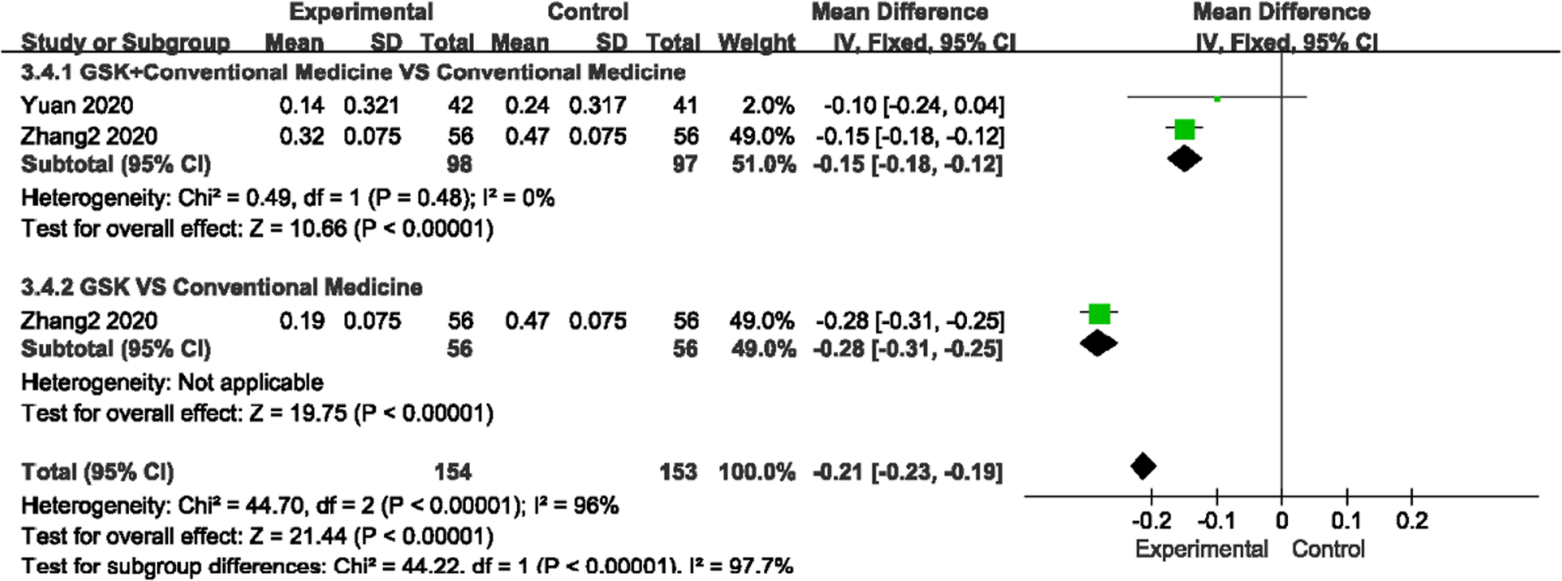
Forest plots of β-CTX levels

For ALP, there was low heterogeneity among the two comparisons (*P* = 0.13, I^2^ = 36%)/(*P* = 0.83, I^2^ = 0%), and for β-CTX, there was no heterogeneity among the studies (*P* = 0.48, I^2^ = 0%), so a fixed-effects model was used to analyze the trials. GSK plus conventional medications (rocaltrol + alendronate sodium, alendronate sodium + Caltrate D, Caltrate D, alfacalcidol and rocaltrol) improved ALP levels compared with conventional medications (MD − 5.56, 95% CI [− 10.08, − 1.04]), but GSK alone did not show an advantage over conventional medications (MD − 1.37, 95% CI [− 13.29, 10.55]). For β-CTX, regardless of whether conventional medications were used (MD − 0.15, 95% CI − 0.18, − 0.12]) or not (MD − 0.28, 95% CI [− 0.31, − 0.25]), GSK had better effects (Fig. 10, 11).

##### Bone glutamyl protein (BGP) and prepeptide of type I procollagen (PINP) levels

**Fig. 12:**
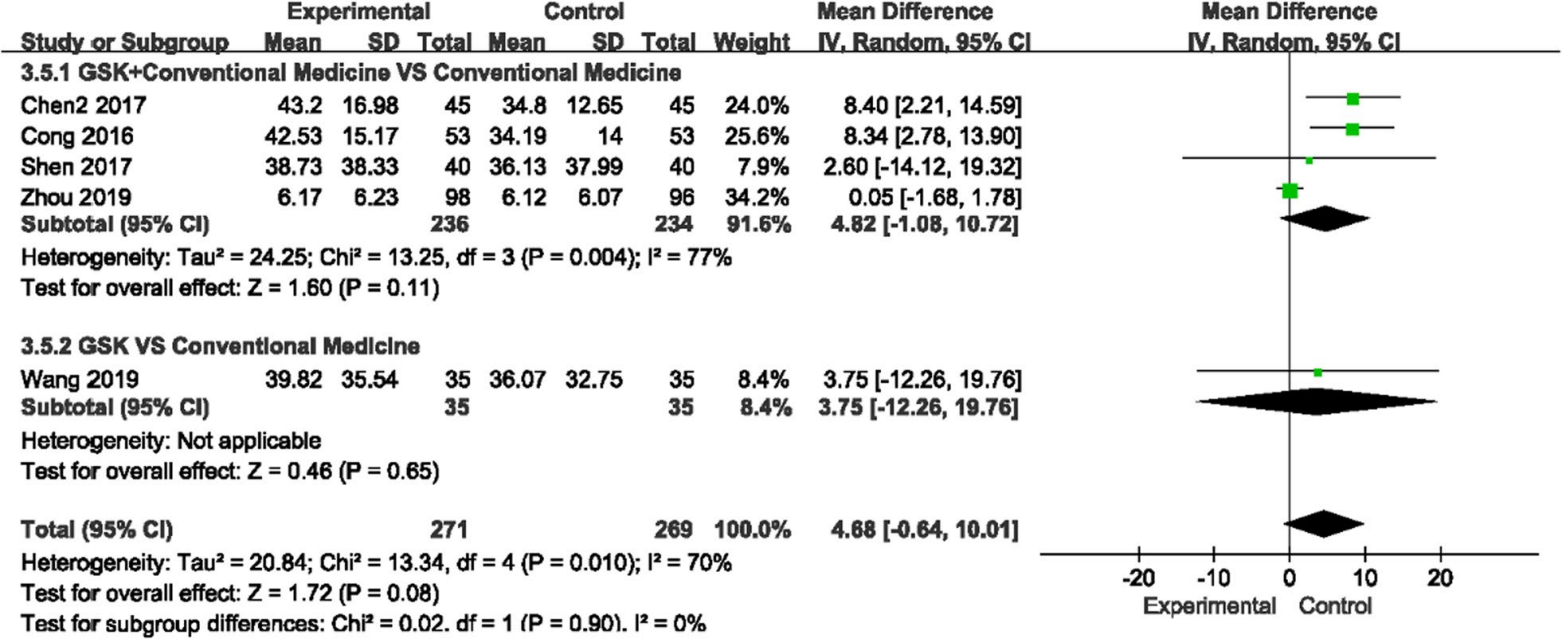
Forest plots of BGP level

**Fig. 13:**
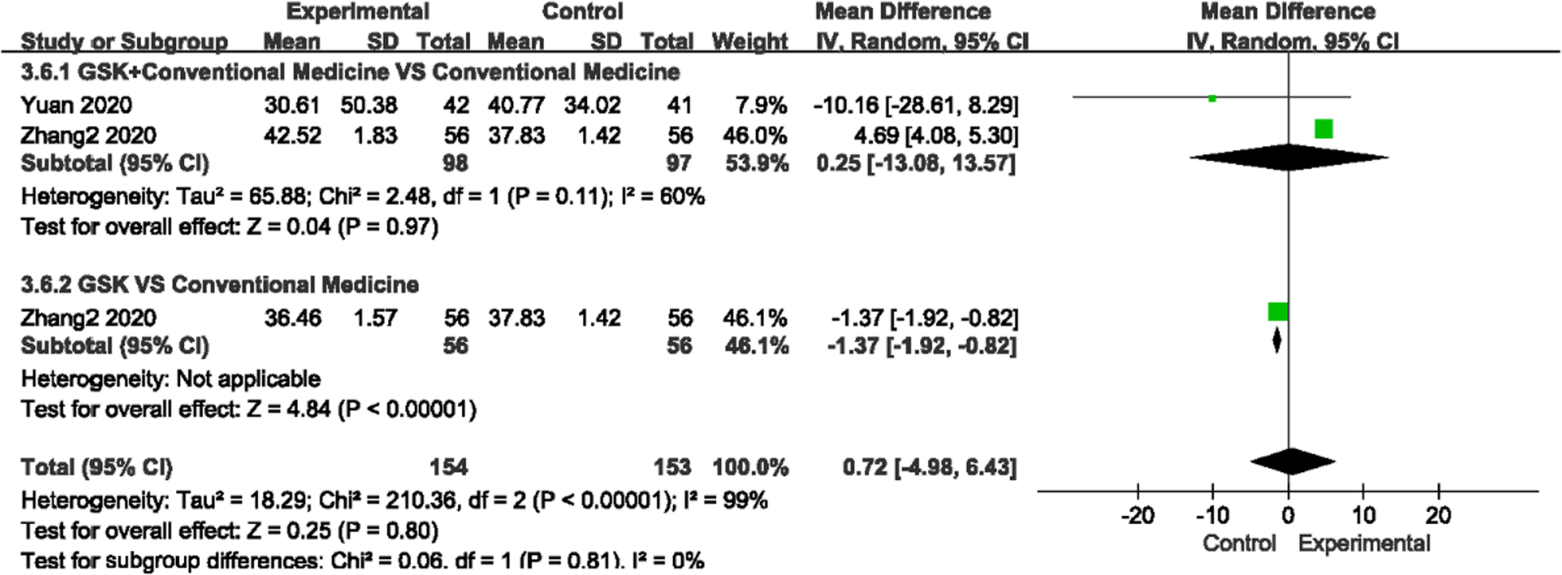
Forest plots of PINP levels

For BGP and PINP, there was high heterogeneity among the studies (*P* = 0.004, I^2^ = 77%)/(*P* = 0.11, I^2^ = 60%); thus, a random-effects model was used to analyze the data (Figs. 12, 13). We found that GSK plus conventional medications did not improve BGP levels more than conventional medications (MD 4.82, 95% CI [− 1.08, 10.27]), nor did GSK alone (MD 3.75, 95% CI [− 12.26, 19.76]). For PINP, the results were conflicting. It was decreased in GSK plus rocaltrol compared to rocaltrol but was higher in GSK plus alendronate sodium + rocaltrol + Caltrate D compared to conventional medications alone. It was also shown that alendronate sodium + rocaltrol + Caltrate D had a better effect than GSK alone on improving the level (MD −1.37, 95% CI [− 1.92, − 0.82]).

#### Adverse events (AE)

**Fig. 14:**
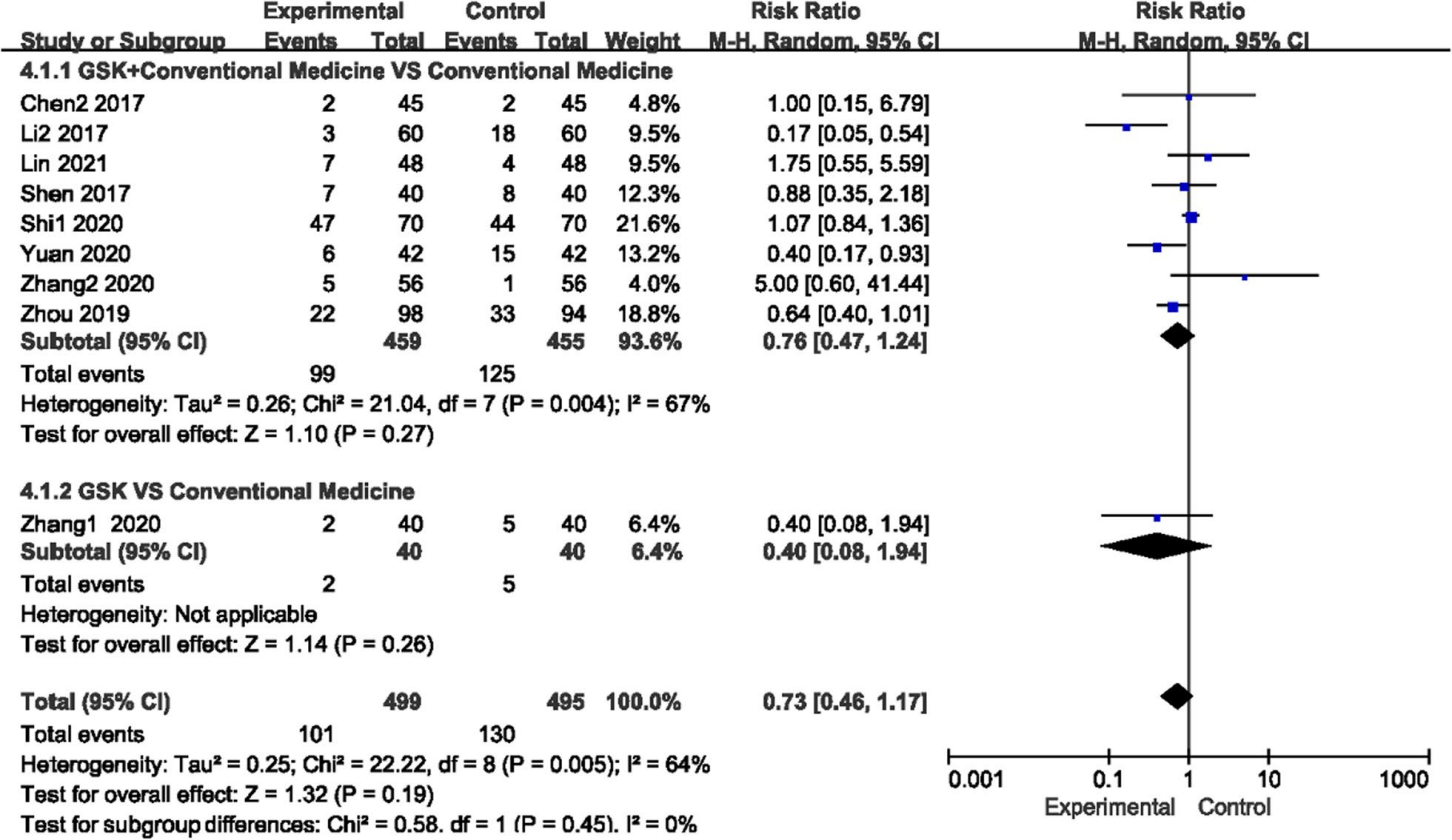
Forest plots of AE

As shown in Fig. 14, there was high heterogeneity among the trials of the GSK-supplemented conventional medication group (or used alone) and the conventional group (*P* = 0.004/0.005, I^2^ = 67%/64%), so a random-effects model was used to analyze the studies. No obvious difference was found in the overall incidence of any adverse events between GSK (used alone (RR 0.40, 95% CI [0.08, 1.94]) or as add-on therapy (RR 0.76, 95% CI [0.47, 1.24])) and conventional medications. The reported adverse events in the GSK group included five cases of headache, eleven cases of losing appetite, eleven cases of flushing, thirty-five cases of gastrointestinal reactions and seven cases of constipation, and those in the conventional medication group included seventeen cases of headache, eleven cases of flushing, twenty-one cases of muscle pain, seventeen cases of fever and twenty-nine cases of gastrointestinal reaction.

### Publication bias assessment

**Fig. 15:**
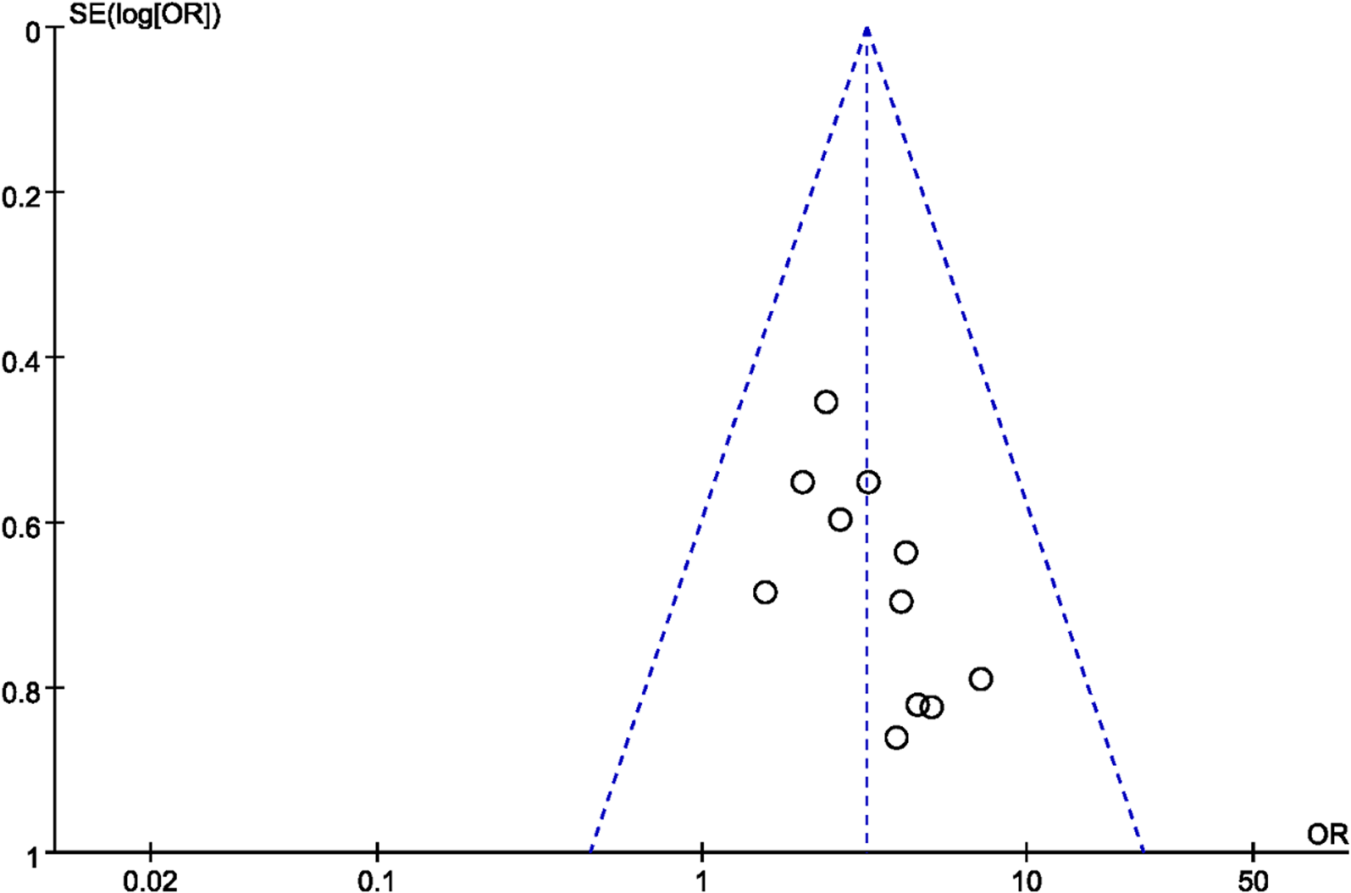
Funnel diagram of OE

**Fig. 16:**
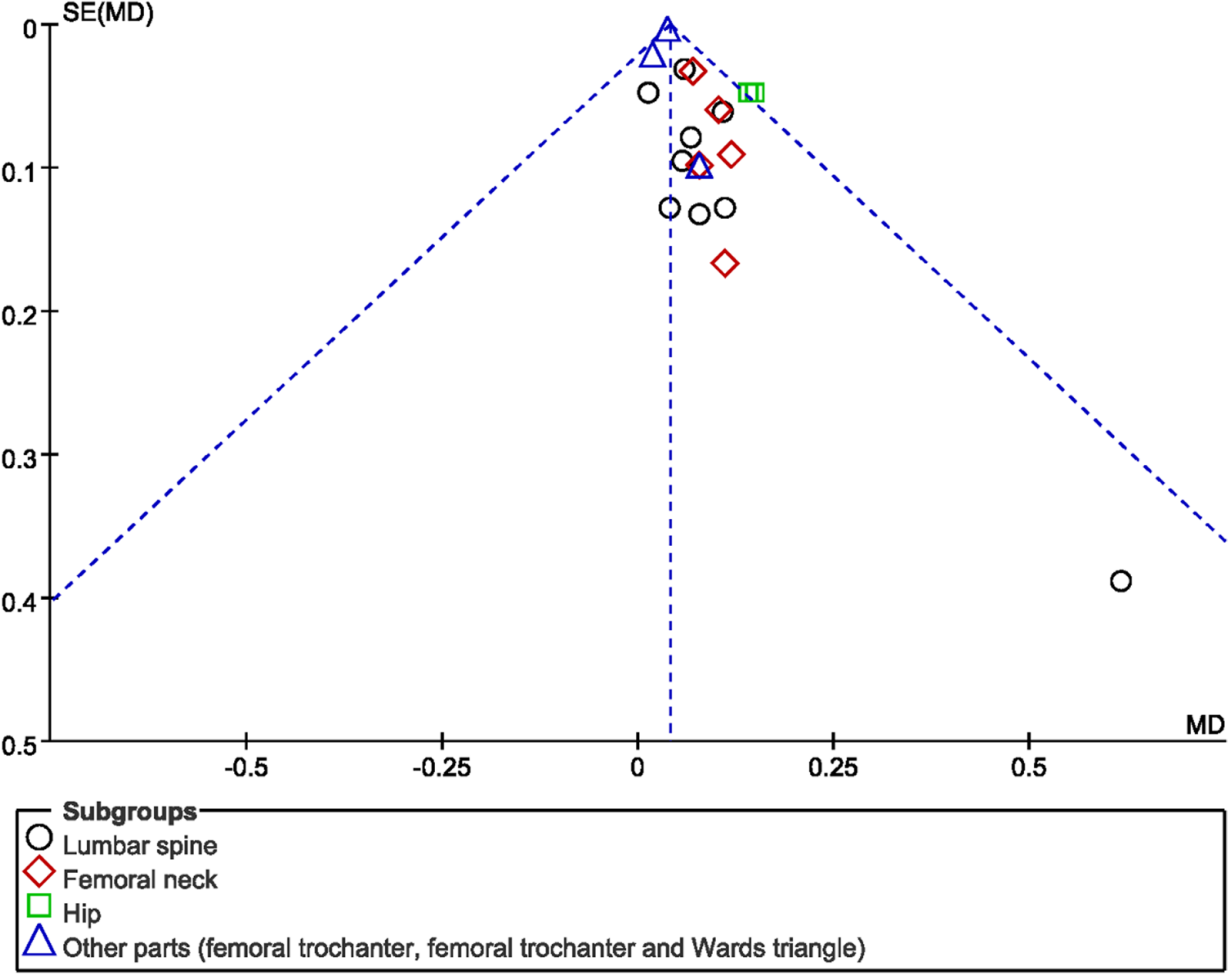
Funnel diagram of BMD

The number of individual outcome index studies included in this systematic evaluation that was more than 10 included OE and bone mineral density, all of which belonged to the comparison of the GSK plus conventional medications group with the conventional medications group, so funnel plots were made to assess their publication bias. As shown in Figs. 15 and 16, the funnel plots of OE were basically symmetrical on the left and right, so there was no publication bias in clinical efficacy; however, the funnel plots of BMD were asymmetrical on the left and right, indicating that there might be publication bias among the studies, probably due to the negative results of some studies not being published.

## Discussion

Generally, the GSK capsule is a safe treatment for POP patients who can help to improve clinical efficacy, regulate bone metabolism and reduce pain as an add-on therapy when compared to conventional drugs such as alfacalcidol, alendronate sodium, salcatonin, nilestriol and vitamin D alone or in combination. Furthermore, GSK capsules plus conventional drugs worked better than conventional drugs alone. It did not have a significant impact on BGP, S-Ca or S-P levels. The results regarding PINP levels were controversial, which might be due to inadequate data.

As a Chinese medicine, the GSK capsule is mainly made of a few Chinese herbs, making its composition difficult to fully detect and analyze. By treating osteoporosis rats with GSK, Lin et al. found that GSK capsules modulated differentially abundant metabolites and proteins involved in nucleotide metabolism, immune processes and general cellular processes to affect bone metabolism and played a significant role in bone protection [52]. Through animal and in vitro cell experiments, Li et al. found that GSK may increase bone mass by promoting bone formation and H-vessel formation and by inhibiting bone resorption, and they believed that these functions may be related to the activity of HIF-1α [53]. Moreover, it was confirmed that treatment of OVX rats with GSK could significantly enhance the BMP-2/Smad signaling pathway by upregulating the expression of BMP-2, p-Smad1, p-Smad5, Osterix and Runx2, and it could also inhibit osteoblast apoptosis by upregulating Bcl-xl and downregulating Bak, suggesting that GSK has a protective effect on promoting bone formation and preventing osteoblast apoptosis. The underlying mechanism may be its regulation of the BMP-2/Smad signaling pathway and the Bcl2 family [54].

A previous meta-analysis reviewed the efficacy and safety of GSK capsules in treating POP [55]. The improvement in S-Ca levels from the GSK group was observed, but the levels of S-P, ALP, BMD and VAS score were found to have no significant difference with conventional medications. Furthermore, the study did not report PINP and β-CTX, which are of vital importance in the progress of bone formation and disintegration. Moreover, the GSK group of the review included both GSK alone and GSK plus conventional medications, which were not specific enough. In contrast to that previous review, there are some advantages in ours: (1) the first meta-analysis on this topic written in English and only fully randomized investigational clinical trials were included, making the results more objective; (2) our outcomes additionally included two indispensable biochemical indicators: PINP and β-CTX, which are important in bone metabolism; (3) our analysis was refined to the comparison of osteoporosis and osteoporosis plus conventional treatment versus conventional treatment, which made the comparison differences more concrete.

However, there are some limitations in our review: (1) all of the studies included in our meta-analysis were in Chinese of medium to low quality, and there was a lack of relevant literature in other languages, which might lead to limitations in scope and reliability of conclusions; (2) the number of comparisons between the GSK group and the conventional treatment group was small, so there was insufficient evidence for the efficacy of osteoporosis alone in the treatment of primary osteoporosis; (3) fracture was the final outcome of osteoporosis development, but none of the included studies used fracture incidence as an outcome indicator, and fracture incidence-related indicators such as β-CTX and PINP were mentioned, but the number of included studies was too small and thus the strength of evidence was insufficient; (4) the studies included were not rigorously implemented or had inconsistent standards for randomization, blinding, allocation concealment, and documentation of outcome indicators, with only one mentioning "double-blind", which may cause an impact on the credibility of the results; (5) the small individual sample sizes of the included trials (19–89 patients) might be insufficient to derive effect estimates; and (6) the wide variety of conventional drugs used in the control group, including different combinations of manufacturers and dosages, made it difficult to analyze them in subgroups and study their efficacy separately.

Clinicians should be aware that the evidence to date for GSK capsules is relatively limited due to the small size of the trials or the high risk of bias. Thus, we are looking forward to future related studies, and there will be more RCTs with large samples and multiple centers. Moreover, higher standards of trial implementation and result recording will be unified to further improve the quality of the study, which will in turn improve the accuracy and credibility strength of the evaluation of the updated system afterwards.

## Conclusion

In our review, it is suggested that the GSK capsule effectively and safely treated primary osteoporosis, while combined with conventional medications, the drug significantly increased bone mineral density, relieved pain and improved bone metabolism-related indicators in patients with primary osteoporosis with better efficacy. However, due to the inclusion of Chinese literature and possible publication bias, the strength of the conclusion still requires more high-quality RCTs.

### Supplementary Information

**Additional file 1**: Protocol of the meta-analysis.

## Data Availability

The data that support the findings of this study are available from the corresponding author.

## Appendix: Search strategy for PubMed

((gushukang[Title/Abstract]) OR ("gushukang" [Supplementary Concept])) AND (((("Osteoporosis"[Mesh]) OR (((((((((((((((((((((Osteoporoses[Title/Abstract]) OR (Osteoporosis, PostTraumatic[Title/Abstract])) OR (Osteoporosis, Post Traumatic[Title/Abstract])) OR (Post-Traumatic Osteoporoses[Title/Abstract])) OR (Post-Traumatic Osteoporosis[Title/Abstract])) OR (Osteoporosis, Senile[Title/Abstract])) OR (Osteoporoses, Senile[Title/Abstract])) OR (Senile Osteoporoses[Title/Abstract])) OR (Osteoporosis, Involutional[Title/Abstract])) OR (Senile Osteoporosis[Title/Abstract])) OR (Osteoporosis, Age-Related[Title/Abstract])) OR (Osteoporosis, Age Related[Title/Abstract])) OR (Bone Loss, Age-Related[Title/Abstract])) OR (Age-Related Bone Loss[Title/Abstract])) OR (Age-Related Bone Losses[Title/Abstract])) OR (Bone Loss, Age Related[Title/Abstract])) OR (Bone Losses, Age-Related[Title/Abstract])) OR (Age-Related Osteoporosis[Title/Abstract])) OR (Age Related Osteoporosis[Title/Abstract])) OR (Age-Related Osteoporoses[Title/Abstract])) OR (Osteoporoses, Age-Related[Title/Abstract]))) OR ("Osteoporosis, Postmenopausal"[Mesh])) OR ((((((((((((((((Perimenopausal Bone Loss[Title/Abstract]) OR (Bone Loss, Postmenopausal[Title/Abstract])) OR (Bone Losses, Postmenopausal[Title/Abstract])) OR (Postmenopausal Bone Losses[Title/Abstract])) OR (Osteoporosis, PostMenopausal[Title/Abstract])) OR (Osteoporoses, PostMenopausal[Title/Abstract])) OR (Osteoporosis, Post Menopausal[Title/Abstract])) OR (Post-Menopausal Osteoporoses[Title/Abstract])) OR (Post-Menopausal Osteoporosis[Title/Abstract])) OR (Postmenopausal Osteoporosis[Title/Abstract])) OR (Osteoporoses, Postmenopausal[Title/Abstract])) OR (Postmenopausal Osteoporoses[Title/Abstract])) OR (Bone Loss, Perimenopausal[Title/Abstract])) OR (Bone Losses, Perimenopausal[Title/Abstract])) OR (Perimenopausal Bone Losses[Title/Abstract])) OR (Postmenopausal Bone Loss[Title/Abstract]))).
