# Supplementary material for: Chinese medicine Gushukang capsule for treating primary osteoporosis: a systematic review and meta-analysis
Source: J Orthop Surg Res. 2023 Nov 8;18:845. doi: 10.1186/s13018-023-04264-9 (PMC10631217; doi:10.1186/s13018-023-04264-9)
Supplement: Supplementary file 1 — Additional file 1: Protocol of the meta-analysis. [file 13018_2023_4264_MOESM1_ESM.pdf]

INPLASY

Chinese patent Medicine Gushukang Capsule for Osteoporosis: A Systematic Review and Meta-Analysis

INPLASY202370023
doi: 10.37766/inplasy2023.7.0023
Received: 07 July 2023
Published: 07 July 2023

Liu, TP[1]; Yao, ML[2]; Zhao, YF[3]; Zhao, SC[4]; Rui, C[5]; Yang, F[6].

Corresponding author:
Tianpeng Liu

2536718065@qq.com

Author Affiliation:
Shaanxi University of Chinese Medicine.

ADMINISTRATIVE INFORMATION

Support - ①the National Natural Science Foundation project (No. 81973889) ②Shaanxi Provincial Education Department Service Local Scientific Research Program: Development of Tai Bai Tong Luo Gel Cream (No.19JC013).

Review Stage at time of this submission - Preliminary searches.

Conflicts of interest - None declared.

INPLASY registration number: INPLASY202370023

Amendments - This protocol was registered with the International Platform of Registered Systematic Review and Meta-Analysis Protocols (INPLASY) on 07 July 2023 and was last updated on 07 July 2023.

INTRODUCTION

Review question / Objective P: Patients with primary osteoporosis. I: Treat the patients with the Chinese patent medicine Gushukang Capsule or combined with coventional western medcine. C: Treat the patients with coventional western medcine alone. O:Primary Outcomes 1. Overall efficacy (OE); 2. Bone mineral density (BMD); 3. Visual analogue score (VAS) for pain. Secondary Outcomes 1. pRocollagen type I N terminal propeptide (PINP); 2. βisomer of C-terminal telopeptide of type I collagen (β-CTX); 3. Alkaline phosphatase (ALP); 4. Serum calcium (S-Ca); 5. Serum phosphorus (S-P); 6. Bone glutamyl protein (BGP); 7. Number and types of adverse reactions. S:RCT trials.

Condition being studied Primary osteoporosis (POP) is a bone metabolic disease characterized by increased bone fragility and fracture risk due to reduced bone mass and destruction of bone tissue microarchitecture (Kanis JA et al., 1994), and is divided into three categories: primary, secondary and idiopathic. Postmenopausal osteoporosis and osteoporosis in the elderly are the most common types of primary osteoporosis in clinical practice (Glaser DL & Kaplan FS,1997). Take China for example, it is a populous country with an ageing population, and some studies predict that by 2050, 25% of the population (about 400 million) will be over 60 years old and the number of people suffering from POP will reach 212 million (Lin X et al., 2015), and the number of POP-related fractures will also increase sharply in the coming decades (Roux C & Briot K, 2020).

METHODS

Search strategy Search China National Knowledge Infrastructure (CNKI), Wan fang database, VIP information, Chinese Biomedical Literature Database (Sino-Med), Web of Science, US National Library of Medicine (PubMed) and

Liu et al. INPLASY protocol 202370023. doi:10.37766/inplasy2023.7.0023 Downloaded from https://inplasy.com/inplasy-2023-7-0023/

Cochrane library from their inception to March 19th, 2023. The following search terms are used individually or combined: “osteoporosis”, “bone loss”, “bone disease”, “Gushukang”, and “randomized controlled trial”. Chinese search terms included “guzhishusong”, “Gushukang”, “suijiduizhaoshiyan”.
A comprehensive search is conducted by adjusting the search strategy according to the different characteristics of the databases in order to improve the completeness of the literature.

Participant or population Patients diagnosed with primary osteoporosis, and the diagnostic criteria refer to "Guidelines for the diagnosis and management of primary osteoporosis(2017)". Chin. J. Osteoporosis (2019) 25(03):281-309. doi:10.19538/j.nk2018020109.

Intervention Treat the patients with the Chinese patent medicine Gushukang Capsule or combined with coventional western medcine.

Comparator Treat the patients with coventional western medcine alone.

Study designs to be included RCT.

Eligibility criteria 1 Inclusion Criteria 1) Blinding is required; 2) Trials with a Jadad score ≥3 could be included. 2 Exclude Criteria1) Original data: duplicate published literature, incomplete study data or type of literature such as review and case report. 2) Study subjects: animal experiments or laboratory studies, patients with secondary osteoporosis or patients with primary osteoporosis combined with severe complications (e.g. fractures). 3) Study type: non-randomized controlled trial. 4) Intervention: containing use of Gushukang capsule in the control group. 5) Study outcome: missing data or obvious errors in the data.

Information sources China National Knowledge Infrastructure (CNKI), Wan fang database, VIP information, Chinese Biomedical Literature Database (Sino-Med), Web of Science, US National Library of Medicine (PubMed) and Cochrane library.

Main outcome(s) Primary Outcomes 1. Overall efficacy (OE); 2. Bone mineral density (BMD); 3. Visual analogue score (VAS) for pain.
Secondary Outcomes1. pRocollagen type Ⅰ N terminal propeptide (PINP); 2. βisomer of C-terminal telopeptide of type Ⅰ collagen (β-CTX); 3. Alkaline phosphatase (ALP); 4. Serum calcium (S-Ca); 5. Serum phosphorus (S-P); 6. Bone glutamyl protein (BGP); 7. Number and types of adverse reactions.

Quality assessment / Risk of bias analysis Cochrane Collaboration's risk of bias assessment tool.

Strategy of data synthesis The data were analyzed using Review Manage. The $\chi 2$ and P values of the study results were tested for heterogeneity. When the value of $P > 0.05$ and $I2 \leq 50\%$, there was no statistical heterogeneity in the results of the studies and a fixed-effects model was selected for statistical analysis, and when the value of $P \leq 0.05$ and $I2 > 50\%$, there was statistical heterogeneity in the results of the studies and a random-effects model was chosen for statistical analysis. Publication bias was tested with inverted funnel plot analysis.

Subgroup analysis For the outcome Bone mineral density (BMD), subgroup studies were analyzed according to different parts of the body.

Sensitivity analysis Sensitivity analysis was performed by Review Manage, and the sensitivity of the article was reflected by the change of the effect after the deletion of one of the articles.

Country(ies) involved China.

Keywords Gushukang capsule, primary osteoporosis, systematic review, bone mineral density, bone metabolism.

Contributions of each author
Author 1 - Tianpeng Liu.
Email: 2536718065@qq.com
Author 2 - Menglin Yao.
Email: 2401141918@qq.com
Author 3 - Yifan Zhao.
Email: 2271522840@qq.com
Author 4 - Shaochuan Zhao.
Email: 604558360@qq.com
Author 5 - Chen Rui.
Email: 1273862260@qq.com
Author 6 - Feng Yang.
Email: yangfengdudu@163.com

Liu et al. INPLASY protocol 202370023. doi:10.37766/inplasy2023.7.0023 Downloaded from https://inplasy.com/inplasy-2023-7-0023/
